# Comparison of CT features of 79 cats with intranasal mass lesions

**DOI:** 10.1177/1098612X21994396

**Published:** 2021-02-19

**Authors:** Sarah Bouyssou, Gawain J Hammond, Caroline Eivers

**Affiliations:** 1 Diagnostic Imaging, University of Glasgow School of Veterinary Medicine, Glasgow, UK; 2 VetCT Telemedicine, Cambridge, UK

**Keywords:** Nasal, mass, neoplasia, computed tomography

## Abstract

**Objectives:**

This retrospective multicentre study compared the CT characteristics of cats diagnosed with intranasal mass lesions to determine if defining imaging features exist between different tumour types and between neoplastic and non-neoplastic lesions.

**Methods:**

The medical records of two institutions were reviewed for cats with CT findings consistent with an intranasal mass lesion with subsequent histopathological examination. For each CT scan the mass location, growth pattern, margin distinction, contrast enhancement pattern and presence of intralesional areas of mineralisation or necrosis were recorded. The presence of facial deformity, the location and type of bone changes, extranasal extension of the mass lesion and the regional lymph nodes size, contrast pattern and hilus visibility were also documented.

**Results:**

Thirty-five cats with nasal lymphoma, 28 cats with non-lymphomatous nasal neoplasia (carcinoma or sarcoma) and 16 cats with inflammatory lesions met the inclusion criteria. Cats with non-lymphomatous nasal neoplasia were more likely to show unilateral nasal changes (odds ratio [OR] 3.9), areas of intralesional calcification (OR infinity) and extension of the mass lesion within the frontal sinus (OR 4.5), while cats suffering from nasal lymphoma were more likely to show a mixed (OR 4.5) and expansile growth pattern (OR 7.8), and a regional lymphadenomegaly (OR 2.4). The CT findings in cats diagnosed with inflammatory mass-like lesions were highly variable and overlapped with findings for nasal neoplasms but were significantly associated with the absence of bony changes to the nasal cavity boundaries (OR 10.2).

**Conclusions and relevance:**

Findings from the current study support the ability of CT to aid in the discrimination of tumour type in cats presented with an intranasal mass lesion.

## Introduction

Nasal tumours account for 1–8.4% of all tumours in cats, and are malignant in >90% of cases.^1,2^ Lymphoma accounts for 26–49% of feline nasal malignancies, followed by epithelial tumours (carcinoma, adenocarcinoma, squamous cell carcinoma [SCC]), and sarcomas are less frequently observed.^1–6^ Feline nasal tumours are locally invasive and generally show a low metastatic rate at the time of diagnosis; however, studies assessing the rate of distant metastasis are lacking.^5–7^ While clinical and diagnostic imaging findings may be highly suggestive of neoplasia, a definitive diagnosis of intranasal neoplasia requires biopsies.^3,8^ Nasal biopsies can be challenging and the samples can be non-representative of the lesion. In dogs, the diagnostic success rate of rhinoscopy-assisted biopsy was 83%, and protracted haemorrhage was an uncommon complication.^9^

Differentiating nasal lymphoma from non-lymphoproliferative tumours is important because of the different treatment strategies.^10–15^ In two previous studies in cats, the CT characteristics of the nasal passages were not useful in discriminating between nasal lymphomas and carcinomas; however, the medial retropharyngeal lymph nodes demonstrated stronger contrast enhancement and more homogeneity in cats suffering from nasal lymphomas than those affected by nasal carcinomas.^16,17^ In humans, several CT characteristics are useful to differentiate nasal lymphomas from SCC. Permeative growth is significantly more frequent with nasal lymphomas than SCCs, whereas destructive growth is significantly more frequent with SCC. A remaining maxillary sinus wall within the tumour is more frequently observed in nasal lymphomas, whereas intratumoral necrosis is more frequently observed in SCC than in nasal lymphoma.^18–20^

Furthermore, causes of an intranasal mass lesion other than neoplasia, such as chronic lymphoplasmacytic rhinitis, fungal rhinitis, chronic nasal foreign body and nasal polyps, have been less commonly reported in cats.^16,17,21–25^

Previous studies investigated specific CT characteristics to discriminate rhinitis from nasal neoplasia in cats.^16,17,23^ However, these studies did not exclusively compare only cases with an identified intranasal mass lesion. To our knowledge, there are no studies in the veterinary literature comparing the CT characteristics of feline nasal neoplasia to inflammatory intranasal masses.

The purpose of this retrospective study was to: (a) determine if CT characteristics could be used to discriminate between nasal lymphoma and non-lymphoproliferative nasal tumours in cats; and (b) determine if CT characteristics could be used to discriminate between sinonasal tumours and inflammatory intranasal mass lesions.

## Materials and methods

This was a retrospective multicentre descriptive case series study. Medical records of the University of Glasgow School of Veterinary Medicine and the teleradiology database of VetCT (VETCT Specialists, St John’s Innovation Centre, Cambridge, UK) were reviewed for cats undergoing CT scans of the head with a diagnosis of an intranasal mass lesion between January 2010 and January 2020. For inclusion, cases must have had intranasal biopsies with histopathological examination. Those cases with inflammatory disease diagnosed on histopathology also required surgical biopsies confirming the diagnosis, or clinical resolution at follow-up. Ethical approval for this study was granted by the Glasgow University Veterinary School Research Ethics committee. Data collected for this study included age, breed and sex.

CT studies at Glasgow University School of Veterinary Medicine were performed with the patient in sternal recumbency under general anaesthesia using a two-slice CT scanner (Siemens Somatom Spirit). The CT scan parameters included helical acquisition, slice thickness 1.0 mm, 130 kVp, 65–100 mAs, field of view ranging from 100 to 150 mm, matrix size 521 × 512, and medium- and high-frequency reconstruction algorithms. When post-contrast series were performed, they were acquired following intravenous administration of 2 ml/kg of an iodinated non-ionic contract agent (Optiray 300 mgI/ml solution for injection; Ioversol). For the patients collected from VetCT medical records, scanning protocols and parameters were variable; however, all studies included a pre- and post-contrast series.

**Figure 1:**
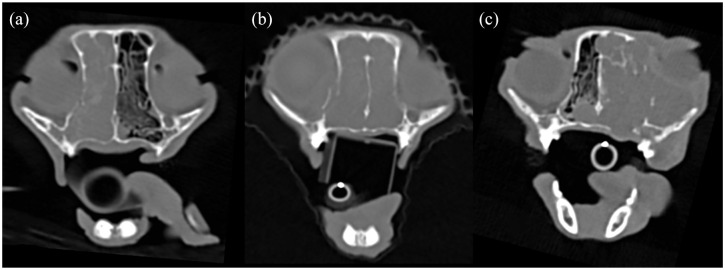
Transverse non-contrast CT images (window level 200 HU, window width 2500 HU) showing three different types of nasal mass growth pattern resulting in paranasal bone lysis. (a) Transverse CT image at the level of the third upper premolar teeth displaying a permeative growth pattern: the mass lesion crosses through the right maxilla bone, does not displace it and leaves most of it in situ; nasal lymphoma was confirmed on histopathology. (b) Transverse CT image at the level of the second upper premolar teeth illustrating an expansile growth pattern: the mass lesion displaces peripherally a portion of the right maxilla bone and extends within the right orbit; nasal lymphoma was confirmed on histopathology. (c) Transverse CT image at the level of the third upper premolar teeth showing a destructive growth pattern: the mass lesion has caused almost complete destruction of the left maxilla and lacrimal bones and extends within the left orbit; note the dorsal and ventral extension of the mass lesion within the right nasal cavity through two nasal septum defects; nasal adenocarcinoma was confirmed on histopathology

CT images were reviewed by a second-year resident in diagnostic imaging (SB) who was blinded to the histopathological findings. Studies were also reviewed by a board-certified radiologist (CE), also blinded to the histopathological findings. Where there were discrepancies between reviewers, a consensus was reached. CT images were reviewed using a commercially available imaging software (ClearCanvas; Synaptive Medical). The data recorded for each study are summarised in Table 1. A mild pattern of enhancement was defined as a difference of less than 50 HU between the pre- and post-contrast images, while a moderate pattern of enhancement was defined as a differential of 50–100 HU between the pre- and post-contrast images, and a strong pattern of enhancement was defined as a difference of more than 100 HU between the pre- and post-contrast images. The presence of intratumoral necrosis or cystic lesions was defined as focal hypodense regions on contrast-enhanced CT images. A permeative tumour was defined as an invasive lesion, which crossed through adjacent bones without displacing them and leaving residual bone in situ. An expansile tumour was defined as a lesion which displaced the surrounding bones peripherally. A destructive-type tumour was defined as a tumour causing total osteolysis of the bone as it grows (Figure 1). When available, CT scans at the time of admission and follow-up of the thorax were also reviewed for the presence of pulmonary nodules, intrathoracic lymphadenopathy and pleural effusion.

**Table 1:** CT features characterised for each patient

Feature	Findings recorded
Laterality	Unilateral, bilateral
Location	Focal/multifocal, diffuse
Margins	Well-defined, ill-defined
Facial deformity	None, exophthalmos, paranasal, parafrontal
Strength of enhancement	Mild, moderate, strong
Pattern of enhancement	Homogeneous, heterogeneous
Presence of intralesional calcification	No, yes
Presence of intralesional necrosis	No, yes
Growth pattern	Turbinate destruction only, permeative, expansive, destructive
Type of bone changes	None, erosion, hyperostosis/sclerosis
Location of bone changes	Nasal bone, maxilla bone, lacrimal bone, palatine bone, vomer bone, frontal bone, zygomatic bone, cribiform plate
Extrasinonasal tumour extension	None, nasopharynx, orbit, intracranial, paranasal, parafrontal
Involvement of the frontal sinus	None, effusion, nasal mass invasion
Regional lymph nodes size	Normal, enlarged
Regional lymph nodes contrast enhancement	Homogeneous, heterogeneous
Regional lymph nodes hilus visibility	Visible, not visible

Statistical analysis was performed by one of the authors (SB) under the supervision of an experienced statistician, using dedicated statistical software package (Minitab Statistical Software; Minitab). Variables examined were those related to the animals (breed, sex, age), location, laterality, presence of facial deformity, type of facial deformity, mass enhancement strength and pattern, presence of internal necrosis or calcification, type of growth pattern, type of bone changes, location of bone changes, extrasinosal extension, involvement of the frontal sinus and regional lymph nodes size, contrast enhancement and visibility of the hilus. Cats with lymphoma vs non-lymphomatous neoplasia, cats with nasal neoplasia vs inflammatory mass, cats with lymphoma vs inflammatory group and cats with non-lymphomatous neoplasia vs inflammatory group were compared. After testing for normal distribution, a *t*-test was used to compare the age between the different groups. Binary logistic regression was used to evaluate differences in the groups regarding sex and neutering status. For breed comparisons, each group was divided into two categories, domestic shorthair or other breeds, and compared using a χ^2^ test. The CT findings were categorised in binomial data and compared by using a χ^2^ test or a Fisher’s exact test when fewer than five patients were present in one category. A critical *P* value of <0.05 was considered significant in all analysis.

## Results

A total of 79 cats met the inclusion criteria; 35 (44%) had nasal lymphoma, 28 (35%) had nasal non-lymphomatous neoplasia (25 carcinomas, three sarcomas) and 16 (20%) had inflammatory mass-like lesions. Inflammatory subtypes included six nasal polyps, four lymphoplasmacytic rhinitis, three fungal rhinitis (two cryptococcosis and one aspergillosis), one mycobacterial nasal infection, one lymphoplasmacytic rhinitis associated with a foreign body, and one combined lymphoplasmacytic rhinitis and aspergillosis. There were 55 domestic shorthair cats, five Siamese, four domestic longhairs, four Maine Coons, two British Shorthairs, one Abyssinian, one Bengal, one Birman, one English Shorthair, one Havana, one Norwegian Forest Cat, one Oriental, one Russian Blue and one Sphynx. Forty-two were neutered male cats, 24 were neutered female cats, eight were entire male cats and five were entire female cats. Cats with nasal lymphoma ranged from 2 to 16 years of age (mean age of 8.3 years), cats with non-lymphomatous neoplasia ranged from 3 to 18 years (mean age of 11.0 years) and cats with inflammatory mass-like lesions ranged from 0.4 to 14 years (mean age of 7.5 years). Demographics for the different categories are shown in Table 2. There were no statistically significant differences between the groups with regard to breed and sex. The cats in the non-lymphomatous neoplasia group were significantly older than the cats in the lymphoma group and the cats in the inflammatory group (*P* = 0.005 and *P* = 0.018, respectively). There was no statistically significant difference in age between the lymphoma and inflammatory groups.

**Table 2:** Distribution of the number of cats, age, sex and breed for the lymphoma, non-lymphomatous neoplasia and inflammatory groups

	Lymphoma (n = 35)	Non-lymphomatous neoplasia (n = 28)	Inflammatory (n = 16)
Age (years)			
Mean	8.3	11	7.5
Range	2–16	3–18	0.4–14
Sex			
Male neutered	17	15	10
Female neutered	13	8	3
Male entire	4	2	2
Female entire	1	3	1
Breed			
Domestic shorthair	24	21	10
Siamese	2	2	1
Domestic longhair	2	1	1
Maine Coon	1	2	1
British Shorthair	1	1	0
Abyssinian	0	1	0
Bengal	1	0	0
Birman	0	0	1
English Shorthair	0	0	1
Havana	1	0	0
Norwegian Forest Cat	0	0	1
Oriental	1	0	0
Russian Blue	1	0	0
Sphynx	1	0	0

The CT findings for each category are summarised in Table 3.

**Table 3:** CT findings for the lymphoma, non-lymphomatous neoplasia and inflammatory groups

	Lymphoma (n = 35)	Non-lymphomatous neoplasia (n = 28)	Inflammatory (n = 16)	Total (n = 79)
Laterality				
Unilateral	19 (54)	23 (82)	11 (69)	53 (67)
Bilateral	16 (46)	5 (18)	5 (31)	26 (33)
Location				
Focal/multifocal	10 (29)	14 (50)	10 (63)	34 (43)
Diffuse	25 (71)	14 (50)	6 (37)	45 (57)
Margins				
Well defined	11 (31)	8 (29)	4 (25)	23 (29)
Ill defined	24 (69)	20 (71)	12 (75)	56 (71)
Facial deformity				
None	17 (49)	16 (57)	12 (75)	45 (57)
Exophthalmos	13 (37)	7 (25)	3 (19)	23 (29)
Paranasal	13 (37)	11 (39)	2 (13)	26 (33)
Parafrontal	2 (6)	1 (4)	1 (6)	4 (5)
Strength enhancement				
Mild	11 (37)	5 (19)	3 (19)	19 (26)
Moderate	14 (43)	15 (58)	10 (62)	35 (54)
Strong	5 (17)	6 (23)	3 (19)	14 (20)
No contrast study	5	2	0	
Pattern enhancement				
Homogeneous	7 (23)	18 (69)	2 (12)	27 (37)
Heterogeneous	23 (77)	8 (31)	14 (88)	45 (63)
No contrast study	5	0	0	
Intralesional calcification				
Absent	35 (100)	23 (82)	14 (88)	72 (91)
Present	0 (0)	5 (18)	2 (12)	7 (9)
Intralesional necrosis				
Absent	22 (73)	20 (77)	15 (94)	57 (79)
Present	8 (27)	6 (23)	1 (6)	15 (21)
No contrast study	5	2	0	
Growth pattern				
Turbinate destruction only	0 (0)	2 (7)	4 (25)	6 (8)
Permeative	25 (71)	16 (57)	9 (56)	50 (63)
Expansile	22 (63)	5 (18)	5 (31)	32 (41)
Destructive	22 (63)	15 (54)	4 (25)	41 (52)
Mixed pattern	25 (71)	10 (36)	5 (31)	40 (51)
Type of bone changes				
Absent	0 (0)	2 (7)	4 (25)	6 (8)
Erosive	35 (100)	26 (93)	12 (75)	57 (72)
Hyperostosis/sclerosis	5 (14)	8 (29)	3 (19)	16 (20)
Location of bone changes				
Nasal	11 (31)	9 (32)	2 (13)	22 (30)
Maxilla	32 (91)	23 (82)	10 (63)	65 (89)
Lacrimal	17 (49)	10 (36)	4 (25)	31 (43)
Palatine	6 (17)	5 (18)	1 (6)	12 (16)
Vomer	14 (40)	7 (25)	6 (38)	27 (37)
Frontal	2 (6)	5 (18)	1 (6)	8 (11)
Zygomatic	3 (9)	4 (14)	0 (0)	7 (10)
Cribiform plate	12 (34)	9 (32)	1 (6)	22 (30)
Tumour extension				
None	1 (3)	2 (7)	2 (13)	5 (6)
Nasopharynx	31 (89)	22 (79)	12 (75)	69 (87)
Orbital	23 (66)	11 (39)	5 (31)	39 (43)
Intracranial	12 (34)	10 (36)	1 (6)	23 (29)
Paranasal	13 (37)	11 (39)	2 (13)	16 (20)
Parafrontal	2 (6)	1 (4)	1 (6)	4 (5)
Involvement of frontal sinus				
Absent	6 (17)	10 (36)	4 (25)	–
Effusion	21 (60)	8 (28)	7 (44)	30 (25)
Mass extension	3 (9)	9 (32)	5 (31)	36 (78)
Material in the sinus but no contrast study	5	1	0	17 (32)
Size regional lymph nodes				
Normal	15 (43)	18 (64)	8 (50)	41 (52)
Enlarged	20 (57)	10 (36)	8 (50)	38 (48)
Enhancement regional lymph nodes				
Homogeneous	21 (70)	20 (77)	14 (88)	55 (76)
Heterogeneous	9 (30)	6 (23)	2 (12)	17 (24)
No contrast study	5	2	0	
Visibility hilus regional lymph nodes				
Visible	15 (43)	18 (64)	10 (62)	43 (54)
Not visible	20 (57)	10 (36)	6 (38)	57 (46)

Data are n (%) unless otherwise indicated

CT of the thorax was available at the time of diagnosis in 39/79 cats (49%; 17 cats with nasal lymphoma, 14 cats with non-lymphomatous nasal neoplasia and eight cats with inflammatory mass-like lesions). Of the 17 cats with nasal lymphoma, soft tissue density pulmonary nodules were detected in three cases (18%), in one case (6%) the cranial mediastinal lymph nodes were enlarged and in an additional case (6%) CT revealed multiple pulmonary nodules associated to a sternal and cranial mediastinal lymphadenopathy and a moderate bilateral pleural effusion. Of the 14 cats with non-lymphomatous neoplasia, pulmonary nodules were detected in three cats (21%) and one case had multiple pulmonary nodules associated with a bilateral moderate pleural effusion. In the benign group, only one case diagnosed with mycobacterial nasal mass had a generalised miliary pattern.Follow-up CT studies of the thorax were available in only 11 cats (14%; six cats with nasal lymphoma, four cats with non-lymphomatous nasal neoplasia and one cat with an inflammatory mass-like lesion). The follow-up period ranged from 21 days to 420 days (average 141 days). Of the six cats with nasal lymphoma, four thoraxes were normal at the time of diagnosis, and remained unremarkable during the follow-up period. One case had multiple soft tissue dense pulmonary nodules at the time of diagnosis which increased in number and size during the follow-up period (420 days). In the last cat with nasal lymphoma, enlarged cranial mediastinal lymph nodes were noted at the time of diagnosis. These lymph nodes remained enlarged and multiple pulmonary nodules developed during the follow-up period (153 days). The four cats with non-lymphomatous neoplasia had a normal thoracic CT at the time of diagnosis and all of them remained unremarkable during the follow-up period (55–293 days, average 181 days). The cat with an inflammatory mass-like lesion (diagnosed as lymphoplasmacytic rhinitis) had a normal thoracic CT at the time of diagnosis and during a follow-up period of 21 days.

**Figure 2:**
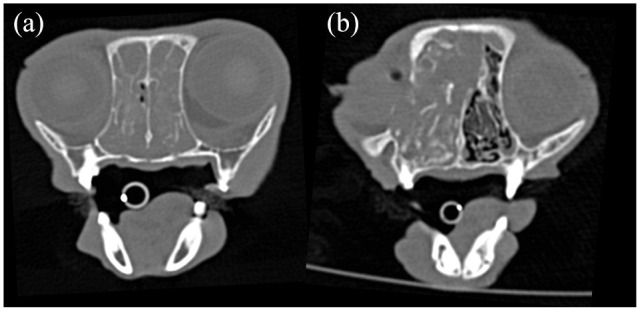
Transverse non-contrast CT images (window level 200 HU, window width 2500 HU) of the nasal cavities at the level of the (a) third and (b) second upper premolar teeth. In (a), the nasal mass extends within both nasal cavities; nasal lymphoma was confirmed on histopathology. In (b), the mass lesion is located within the right nasal cavity, extends mildly within the right periorbital region, displaces the nasal septum to the left and slightly invades the dorsal portion of the left nasal cavity; nasal adenocarcinoma was confirmed on histopathology

**Figure 3:**
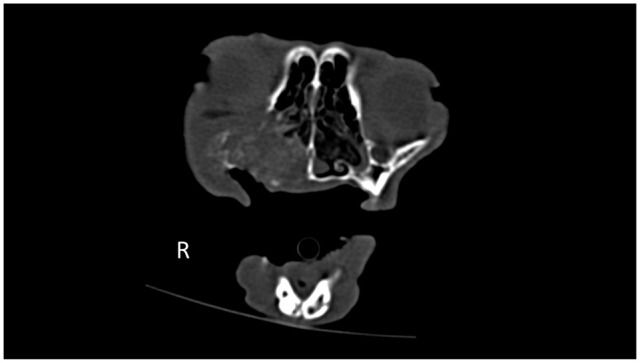
Transverse non-contrast CT image (window level 450 HU, window width 1500 HU) at the level of the fourth upper premolar teeth illustrating the presence of intralesional calcification. Note the destructive growth pattern affecting the right maxilla, palatine and zygomatic bones. Nasal squamous cell carcinoma was confirmed on histopathology

**Figure 4:**
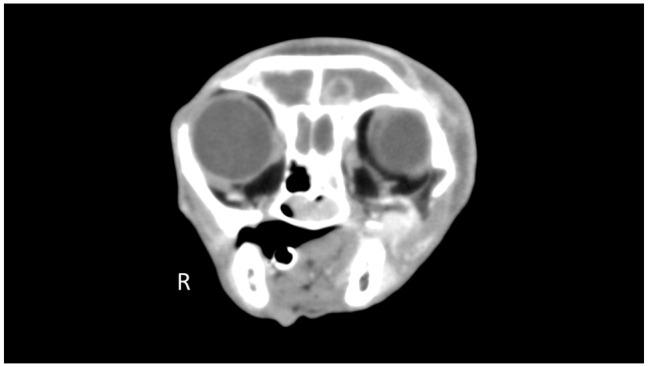
Transverse post-contrast (delayed phase) CT image (window level 30 HU, window width 320 HU) at the level of the frontal sinuses. The caudodorsal extremity of the nasal mass extends within the fluid-filled left frontal sinus. Note also the extension of the mass lesion within the nasopharynx. Nasal adenocarcinoma was confirmed on histopathology

Some imaging characteristics were helpful in differentiating nasal lymphoma from non-lymphomatous nasal neoplasia. Cats with non-lymphomatous nasal neoplasia were more likely to show unilateral nasal changes (odds ratio [OR] 3.9, 95% confidence interval [CI] 1.20–12.53; *P* = 0.02) (Figure 2), intralesional calcification (OR infinity; *P* = 0.014) (Figure 3) and extension of the mass lesion within the frontal sinus (OR 4.5, 95% CI 1.07–18.92; *P* = 0.03) (Figure 4). Cats with nasal lymphoma were more likely to show a mixed growth pattern (OR 4.5, 95% CI 1.55–13.06; *P* = 0.006), an expansile growth pattern (OR 7.8, 95% CI 2.38–25.47; *P* = 0.003) and a regional lymphadenomegaly (OR 2.4, 95% CI 0.86–6.67; *P* = 0.018).

When both types of nasal neoplasia were compared together with the inflammatory group, only the absence of bone changes was correlated with the presence of a non-neoplastic nasal mass (OR 10.2, 95% CI 1.67–61.92; *P* = 0.013).

After comparing the lymphoma group with the inflammatory group, the presence of nasal lymphoma was more likely when the mass lesion occupied the entire nasal cavity (OR 4.2, 95% CI 1.19–14.54; *P* = 0.021). A benign process was more likely in the absence of bony changes to the nasal cavity boundaries (OR infinity; *P* = 0.007) and when the mass lesion extended into the frontal sinus (OR 4.1, 95% CI 0.83–20.14; *P* = 0.039).

There were no useful imaging features in differentiating non-lymphomatous nasal neoplasia to inflammatory mass-like lesions.

## Discussion

This is the first study conducted in a large cohort of cats with an intranasal mass to investigate specific CT characteristics to aid discrimination of nasal neoplasia from inflammatory processes and nasal lymphoma from non-lymphomatous nasal neoplasia.

Unilateral nasal involvement (OR 3.9) and extension of the mass lesion within the frontal sinus (OR 4.5) were more frequently and statistically significantly associated with non-lymphomatous tumours (carcinomas and sarcomas) compared with nasal lymphoma. Interestingly, no case diagnosed with nasal lymphoma showed signs of intralesional calcification, while it was observed in a few cases with non-lymphomatous neoplasia. Furthermore, lymphoma was more often associated with mixed (OR 4.5) and expansile (OR 7.8) growth pattern and regional lymphadenomegaly (OR 2.4).

Several studies have investigated specific CT characteristics to discriminate rhinitis from nasal neoplasia and to discriminate different types of nasal neoplasia in cats.^16,17^ One retrospective assessment of CT imaging in 62 cats with sinonasal disease showed that osteolysis of the paranasal bones, moderate-to-severe turbinate destruction, lysis of the nasal septum, the presence of a homogeneous space-occupying mass and extension of the disease process into the orbit or facial soft tissues may lead to a diagnosis of nasal neoplasia vs rhinitis but were not pathognomonic. This same study also identified some trends to differentiate different tumour tissue types: severe lysis of the turbinates and paranasal bones was more frequently associated with carcinoma and lymphoma; paranasal bone destruction involving adenocarcinoma was often confined to the caudal nasal cavity; and carcinoma often resulted in lysis rostrally and caudally and extended into the orbit, whereas lymphoma was often associated to subtle nasal changes.^16^ Another study evaluating the clinical characteristics and CT findings in 43 cats diagnosed for sinonasal disease found that unilateral lysis of ethmoturbinates, unilateral lysis of the dorsal and lateral maxilla, lysis of the vomer bone and ventral maxilla, bilateral lysis of the orbital lamina, unilateral abnormal soft tissue/fluid in the sphenoid sinus, frontal sinus and/or retrobulbar space were CT characteristics associated with nasal neoplasia.^23^ An additional study sought to determine whether CT characteristics of nasal passages and medial retropharyngeal lymph nodes (MRPLN) could be used to distinguish neoplasia from rhinitis. They found that CT characteristics significantly associated with neoplasia included abnormal MRPLN hilus, paranasal bone lysis, turbinate lysis, mass, MRPLN height asymmetry and decreased MRPLN precontrast heterogeneity. This study also attempted to discriminate the different tumoral types and showed that nasal lymphoma was significantly associated with abnormal perinodal fat and loss of visibility of the hilus, and all cats with nasal lymphoma had at least one of these abnormalities, while nasal carcinoma had abnormal perinodal fat or loss of hilus. In addition, most cats with nasal lymphoma had enlarged medial retropharyngeal lymph nodes and a greater degree of contrast medium enhancement.^17^

The results of our study differ from these previous publications as we defined several nasal passage CT characteristics which differed between nasal lymphoma and non-lymphomatous neoplasia such as the laterality, the growth pattern, the presence of intralesional calcifications and the extension of the mass lesion within the frontal sinus. These additional findings could be explained by the greater number of cases with nasal neoplasia included in our study, the additional CT features evaluated, the method of comparison (lymphoma vs carcinoma and sarcoma together) and the fact that only cats with a nasal mass were included in this study. A significantly greater number of cats in the current study with nasal lymphoma had enlarged regional lymph nodes when compared with those with non-lymphomatous neoplasia.^17^ There was not, however, any significant difference regarding the loss of visibility of the hilus and the strength of contrast enhancement. This could also be due to the variation in study protocols resulting from the multicentre collection of cases.

With the exception of sinonasal neoplasia, other causes of intranasal masses in cats are uncommon and include chronic lymphoplasmacytic rhinitis, fungal rhinitis, chronic nasal foreign body and nasal polyps.^16,17,21–25^ No previous studies have been reported in the veterinary literature to compare specifically the CT features of intranasal inflammatory masses to nasal neoplasia in cats. Only a few cases were recruited in our study and included numerous subtypes such as nasal polyps, lymphoplasmacytic rhinitis, fungal rhinitis, mycobacterial nasal infection, lymphoplasmacytic rhinitis associated with chronic foreign body and combined lymphoplasmacytic rhinitis and aspergillosis. As expected, the CT findings in the inflammatory group were highly variable in the current study and many of the CT features associated with nasal neoplasia overlapped with those found with these inflammatory processes. When compared with all types of neoplasia and nasal lymphoma alone, the absence of bone involvement of the nasal passage boundaries was significantly greater within the inflammatory group (OR 10.2 and infinity, respectively). In addition, when compared with nasal lymphoma alone, the incidence of mass extension within the frontal sinus was significantly greater within the inflammatory group (OR 4.1), while the nasal lymphomas were more likely to occupy the entire nasal cavity (OR 4.2). There were, however, no pathognomonic imaging features that could differentiate non-lymphomatous nasal neoplasia alone to inflammatory mass lesions.

Nasal lymphoma (55.6%) was the most common sinonasal neoplasm and the mean age of cats with nasal neoplasia was 10 years.^2,3,5,13,16,17,23^ In the current study, cats with non-lymphomatous neoplasia (mean age 11 years) were significantly older than the cats with nasal lymphoma (mean age 8.3 years), and the mean age of the cats diagnosed with neoplasia was not greater than the mean age with inflammatory mass lesions. In addition, males and females were equally represented in each group.

Feline sinonasal tumours have previously been described as locally invasive and associated with a low metastatic rate at the time of diagnosis; however, studies assessing the rate of distant metastasis are lacking.^5–7^ In the current study, CT of the thorax was performed in 17 cats with nasal lymphoma (48.6%) and 14 cats with non-lymphomatous nasal neoplasia (50%) at the time of diagnosis. Of the 17 cases with nasal lymphoma, pulmonary nodules, intrathoracic lymphadenopathy and pleural effusion were identified in four (24%), two (12%) and one (6%) cases, respectively. Of the 14 cats with non-lymphomatous neoplasia, pulmonary nodules and pleural effusion were detected in five (36%) and one (7%) cats, respectively. Only a few follow-up CT studies of the thorax were performed during variable periods ranging from 21 days to 420 days (average 141 days), with one case of nasal lymphoma developing pulmonary nodules 153 days after the initial diagnosis. These results illustrate the importance of screening cats with nasal neoplasia for distant or unrelated metastatic disease. Although finding pulmonary nodules, pleural effusion and enlarged lymph nodes in a patient with malignant neoplasia is suggestive of metastasis, other aetiologies are possible, and no histological correlation between the thoracic changes and the nasal pathology was reached in the cases in this study.

There are limitations to this study, which mainly relate to its retrospective nature. The CT examinations were acquired using multiple scanners with different acquisition parameters, slice thickness and contrast administration techniques according to institutional protocols, which may have altered the diagnostic quality and the attenuation values measured. The small sample size of cats affected by inflammatory lesions, the low number of patients present in several subcategories and the number of statistical tests performed are additional limitations that could have affected the results of this study. Finally, intranasal biopsy samples can potentially be non-representative of a lesion, which is a significant conundrum in clinical practice. For this reason, intranasal mass lesions in this study were only presumed to be inflammatory based on histopathology of biopsies if additionally confirmed by either a surgical biopsy or resolution of a patient’s clinical signs. This criteria, however, is not perfect and it is possible that some of the lesions presumed to be inflammatory could have actually been neoplastic in nature with non-representative biopsies.

## Conclusions

Findings from the current study support the ability of CT to aid in the discrimination of tumour type (lymphoma vs non-lymphomatous neoplasia) in cats presented with an intranasal mass lesion. Unilateral nasal involvement (OR 3.9) and extension of the mass lesion within the frontal sinus (OR 4.5) were more frequently associated with non-lymphomatous tumours than with nasal lymphoma. The presence of intralesional calcifications was only observed in cats with non-lymphomatous nasal neoplasia. Lymphoma was more often associated with a mixed (OR 4.5) and expansile (OR 7.8) growth pattern, and regional lymphadenomegaly (OR 2.4). The CT findings in cats diagnosed with inflammatory intranasal mass lesions were highly variable and overlapped with those found with nasal neoplasia; however, the absence of bone involvement of the nasal cavity boundaries is significantly associated with a benign process. None of these findings, however, was pathognomonic for any category and nasal biopsies remain recommended for a definitive diagnosis.
